# CRISPR/Cas9 Mutagenesis of *UL21* in Multiple Strains of Herpes Simplex Virus Reveals Differential Requirements for pUL21 in Viral Replication

**DOI:** 10.3390/v10050258

**Published:** 2018-05-15

**Authors:** Renée L. Finnen, Bruce W. Banfield

**Affiliations:** Department of Biomedical and Molecular Sciences, Queen’s University, Kingston, ON K7L 3N6, Canada; renee.finnen@queensu.ca

**Keywords:** HSV, *UL21*, CRISPR/Cas9

## Abstract

Studies from multiple laboratories using different strains or species of herpes simplex virus (HSV) with deletions in *UL21* have yielded conflicting results regarding the necessity of pUL21 in HSV infection. To resolve this discrepancy, we utilized CRISPR/Cas9 mutagenesis to isolate pUL21 deficient viruses in multiple HSV backgrounds, and performed a side-by-side comparison of the cell-to-cell spread and replication phenotypes of these viruses. These analyses confirmed previous studies implicating the involvement of pUL21 in cell-to-cell spread of HSV. Cell-to-cell spread of HSV-2 was more greatly affected by the lack of pUL21 than HSV-1, and strain-specific differences in the requirement for pUL21 in cell-to-cell spread were also noted. HSV-2 strain 186 lacking pUL21 was particularly crippled in both cell-to-cell spread and viral replication in non-complementing cells, in comparison to other HSV strains lacking pUL21, suggesting that the strict requirement for pUL21 by strain 186 may not be representative of the HSV-2 species as a whole. This work highlights CRISPR/Cas9 technology as a useful tool for rapidly constructing deletion mutants of alphaherpesviruses, regardless of background strain, and should find great utility whenever strain-specific differences need to be investigated.

## 1. Introduction

The *Alphaherpesvirinae* are a subfamily of the *Herpesviridae* that include the human pathogens herpes simplex virus (HSV)-1 and HSV-2. The *UL21* gene encodes a multifunctional protein that is conserved amongst the alphaherpesviruses. The UL21 protein (pUL21) is a component of the tegument, a compartment of the herpesvirus virion located between the capsid and the envelope. Tegument components are delivered to the cell cytoplasm immediately upon viral entry, where they have the opportunity to promote viral infection prior to de novo viral gene expression. In the case of both HSV-1 and HSV-2, pUL21 plays an as yet undefined role early in infection that promotes the initiation of viral gene expression [1,2]. As HSV pUL21 can associate with both capsids [3] and microtubules [4], it has been proposed that pUL21 may directly facilitate the transport of incoming capsids along microtubules to the nucleus, and that delayed capsid transport in the absence of pUL21 results in the delay of viral gene expression [1].

HSV pUL21 is also known to play multiple roles late in infection. In HSV-2, pUL21 is required for efficient translocation of capsids from the nucleus to the cytoplasm, where final virion envelopment and maturation takes place. Herpesvirus capsids are assembled within the nucleus, and are too large to exit the nucleus through nuclear pores. Instead, capsids containing viral genomic DNA undergo primary envelopment at the inner nuclear membrane (INM) followed by de-envelopment at the outer nuclear membrane (ONM), to relocate from the nucleus to the cytoplasm. In cells infected with HSV-2 lacking pUL21, capsids accumulated in the nucleoplasm and were rarely observed in the cytoplasm or in the perinuclear space between the INM and the ONM, consistent with a role for pUL21 in nuclear egress [2]. Accumulation of capsids in the nucleoplasm was not observed in a recent analysis of cells infected with HSV-1 lacking pUL21, however, an increase in the incidence of empty capsids (lacking viral genomic DNA) in both the nucleoplasm and the cytoplasm was noted [5]. Finally, roles for HSV-1 pUL21 have been described in events occurring later in the infectious cycle. In the cytoplasm, pUL21 forms a complex with the tegument proteins pUL16 and pUL11 on the cytoplasmic tail of the viral glycoprotein, gE [6,7,8]. This complex is required for efficient glycosylation of gE and its trafficking to the cell surface [7]. Furthermore, deletion of *UL21* from HSV-1 results in the failure of nascent virions to incorporate pUL16 into the tegument [3]. Crystal structures of both the amino and carboxy termini of HSV-1 pUL21 have been determined [9,10] however, both structures are unique, and thus, have not provided further insight into the multiple roles attributed to pUL21 during HSV infection. Intriguingly, during the isolation of the C-terminal domain of pUL21 for crystallization studies, it was noted that this domain co-purified with bacterial RNA, suggesting that pUL21 may have RNA binding capacity [10].

As pUL21 plays multiple roles in HSV infection, it is not surprising that pUL21 is required for optimal viral replication. What is surprising is that the strictness of this requirement differs between HSV-1 and HSV-2, yet the pUL21s of these distinct virus species are roughly 84% identical. When either homologous recombination or *en passant* mutagenesis of a bacterial artificial chromosome (BAC) was used to introduce a deletion into *UL21* of HSV-1 strains F or KOS, the resulting viruses were able to replicate in the absence of complementation, but had growth deficiencies in non-complementing cells, ranging from 3- to 100-fold, and also produced smaller plaques in non-complementing cells [1,5,11]. By contrast, when BAC *en passant* mutagenesis was used to introduce a deletion into *UL21* of HSV-2 strain 186, the resulting virus was extremely crippled, due primarily to its nuclear egress defect, and required complementation to replicate [2]. Thus, there are discrepancies regarding the necessity of pUL21 in HSV replication: pUL21 is essential in the case of HSV-2 and non-essential in the case of HSV-1. What might explain these discrepancies? One possibility is that the results obtained with the single strain of HSV-2 studied by Le Sage et al., strain 186, were not representative of the HSV-2 species as a whole. Another possibility is that pUL21 has species-specific functions during the replication of HSV-2 and HSV-1.

The goals of this study were to establish whether the phenotype of other HSV-2 strains carrying a deletion in *UL21* resembled strain 186, and to re-examine whether phenotypic differences exist between HSV-1 and HSV-2. To achieve these goals, CRISPR/Cas9 mutagenesis was used to engineer a panel of *UL21* deletion mutants in multiple HSV strains. Comparative analysis of cell-to-cell spread and replication kinetics of this panel of *UL21* deletion mutants in non-complementing cells revealed both strain-specific and species-specific differences in the requirement for pUL21 in HSV replication.

## 2. Materials and Methods

### 2.1. Cells and Viruses

African green monkey kidney cells (Vero) and murine L cell fibroblasts were maintained in Dulbecco’s modified Eagle’s medium (DMEM) supplemented with 10% fetal bovine serum (FBS) in a 5% CO_2_ environment. L cells, 293T cells, and HaCaT cells that stably produce pUL21 of HSV-2 strain 186 (referred to herein as L21, 293T21 and HaCaT21), were similarly maintained. 293T21 and HaCaT21 cells were isolated by retroviral transduction using an amphotropic Phoenix-Moloney murine leukemia virus system, as described previously for the isolation of L21 cells [2]. All viruses used in this study were propagated in either L21 or HaCaT21 cells. HSV-2 strains SD90e, HG52, and 186 were acquired from Dr. D. M. Knipe (Harvard University), Dr. D. J. McGeoch (MRC Virology Unit, University of Glasgow), and Dr. Y. Kawaguchi (University of Tokyo), respectively; HSV-1 strains KOS and F were acquired from Dr. L. W. Enquist (Princeton University). Infection times, reported in hours post infection (hpi) or days post infection (dpi), refer to the length of time following a one hour inoculation period.

### 2.2. Construction of UL21 Guide RNA Expression Plasmids

**Figure 1:**
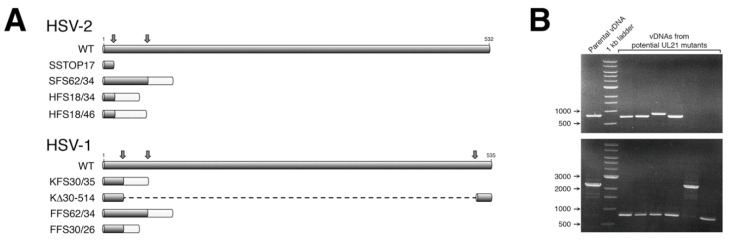
Construction of herpes simplex virus (HSV) *UL21* mutants by CRISPR/Cas9 mutagenesis. (**A**) Truncated *UL21* proteins encoded by CRISPR/Cas9 mutants. Dark grey bars indicate wild type protein; light grey bars indicate non-UL21 amino acids; vertical arrows indicate gRNA-directed cleavage sites within UL21. In the mutant nomenclature, the first letter indicates the parent strain (S = SD90e, H = HG52, K = KOS, F = F); the type of lesion introduced into *UL21* is indicated next (STOP = premature stop codon; FS = frameshift, Δ = in-frame deletion); numbers immediately after the lesion description indicate *UL21* codon coordinates; numbers after the backslash indicate the number of non-UL21 amino acids added as a result of frameshifting. (**B**) Example of a PCR screen of potential HSV *UL21* mutants. PCR products generated from HSV-1 KOS parental vDNA template or vDNA templates prepared from individual plaques arising from co-transfection of KOS vDNA along with two gRNAs designed to introduce small deletions (top panel) or large deletions (bottom panel) into the KOS *UL21* gene were separated on a 0.8% agarose gel and stained with Midori Green. DNA molecular size markers in bp are indicated on the left.

Guide RNAs (gRNAs), used for CRISPR/Cas9 mutagenesis, were expressed from plasmid pX330-U6_Chimeric_BB-CBh-hSpCas9, a gift from Feng Zhang, the Broad Institute of MIT, (Addgene plasmid 42230 [12]). To construct *UL21*-specific gRNA expression plasmids, the top-strand oligonucleotide was annealed to the corresponding bottom-strand oligonucleotide (Table 1), and the double-stranded DNA product was ligated into pX330-U6_Chimeric_BB-CBh-hSpCas9 plasmid that had been digested with *Bbs*I. Two gRNAs were designed and utilized in our initial mutagenesis of HSV-2 *UL21* (Figure 1A). For our subsequent mutagenesis of HSV-1 *UL21*, three gRNAs were designed and utilized (Figure 1A), allowing both small and large deletions to be introduced into the *UL21* locus (Figure 1B).

### 2.3. CRISPR/Cas9 Mutagenesis of the UL21 Locus

Viral genomic DNA (vDNA) of each strain (SD90e, HG52, KOS, or F) was purified as described previously [13]. Subconfluent monolayers of 293T21 cells growing in 100 mm dishes were co-transfected with 16 μg of vDNA, and 1 μg each of two *UL21* gRNA expression plasmids, using a calcium phosphate co-precipitation method [14]. Twenty-four hours after transfection, the culture medium was replaced with semisolid medium containing 1% methyl cellulose, to allow for plaque formation. Five to six days later, individual plaques were picked and collected in 300 μL of medium. Picked plaque solutions were used to infect monolayers of L21 cells growing in 6-well dishes. Viral DNA was isolated from a portion of the infected L21 monolayers using a modified Hirt lysis protocol [15], and used as template for PCR. The *UL21* loci from viruses bearing mutations were sequenced in their entirety to determine the precise nature of the mutation introduced.

### 2.4. Cell-to-Cell Spread Analysis

To analyze macroscopic plaque formation, monolayers of Vero cells were infected with equivalent numbers of plaque forming units, overlaid with semisolid medium containing 1% methyl cellulose, then fixed and stained with 70% methanol containing 0.5% methylene blue at 72 hpi. To analyze cell-to-cell spread quantitatively, monolayers of Vero cells growing on glass bottom dishes (MatTek, Ashland, MA, USA) were infected with equivalent numbers of plaque forming units. At 24 hpi, infected monolayers were fixed with 4% paraformaldehyde and stained for the presence of the HSV kinase Us3, as described previously [16]. Images of plaques were captured on an Olympus FV1000 laser scanning confocal microscope using a 10× objective or on a Nikon TE200 inverted epifluorescence microscope using a 10× objective and a cooled CCD camera. To quantify these results, the numbers of pixels in the area of each plaque were counted using Image-Pro 6.3 software (Media Cybernetics, Bethesda, MD, USA). Results shown were derived from 40 distinct plaques per strain. Single infected cells were not included in this quantification.

### 2.5. Virus Replication Analysis

Monolayers of Vero cells in 6-well dishes (approximately 5 × 10^5^ cells per well) were infected at a multiplicity of infection (MOI) of 0.1. All infections were performed in triplicate. After a one hour inoculation period, monolayers were treated for 2 min at 37 °C with low pH citrate buffer (40 mM Na citrate, 10 mM KCl, 0.8% NaCl) to inactivate extracellular virions, washed once with medium, then incubated at 37 °C. Infected monolayers were harvested by scraping cells and medium together at 0, 24, 48, 72, and 96 hpi and stored at −80 °C. Samples were subjected to two freeze/thaw cycles, sonicated briefly in a chilled cup-horn sonicator, and cellular debris removed from the lysate by brief centrifugation at 3000 rpm in a microcentrifuge. The virus titers of the clarified lysates were determined in duplicate by plaque assay on L21 cells.

### 2.6. Immunological Reagents

Rat polyclonal antiserum against HSV-2 Us3 [16] was used for indirect immunofluorescence microscopy at a dilution of 1:500; rat polyclonal antiserum against HSV-2 pUL21 [2] was used for Western blotting at a dilution of 1:600; mouse monoclonal antibody against HSV ICP27 (Virusys, Taneytown, MD, USA) was used for Western blotting at a dilution of 1:500; mouse monoclonal antibody against β-actin (Sigma, St. Louis, MO, USA) was used for Western blotting at a dilution of 1:2000; Alexa Fluor 488 conjugated donkey anti-rat IgG, (Molecular Probes, Eugene, OR, USA) was used for indirect immunofluorescence at a dilution of 1:500; horseradish peroxidase-conjugated goat anti-mouse IgG (Sigma, St. Louis, MO, USA) was used for Western blotting at a dilution of 1:10,000; horseradish peroxidase-conjugated goat anti-rat IgG (Sigma, St. Louis, MO, USA) was used for Western blotting at a dilution of 1:80,000.

### 2.7. Preparation and Analysis of Whole Cell Lysates

To prepare whole cell lysates of infected cells for Western blot analyses, cells were washed with cold phosphate buffered saline (PBS), then scraped into cold PBS containing protease inhibitors (Roche) plus 5 mM sodium fluoride (New England Biolabs, Ipswich, MA, USA) and 1 mM sodium orthovanadate (New England Biolabs, Ipswich, MA, USA) to inhibit phosphatases. Harvested cells were transferred to a 1.5 mL microfuge tube containing 3× sodium dodecyl sulphate-polyacrylamide gel electrophoresis (SDS-PAGE) loading buffer. The lysate was repeatedly passed through a 28 1/2-gauge needle to reduce viscosity, and then heated at 100 °C for 5 min. For Western blot analysis, 10 to 20 μL of whole cell lysate was electrophoresed through SDS 8% polyacrylamide gels. Proteins were transferred to polyvinylidene fluoride membranes (Millipore, Billerica, MA, USA), blocked with 3% bovine serum albumin, then probed with appropriate dilutions of primary antibody followed by appropriate dilutions of horseradish peroxidase conjugated secondary antibody. The membranes were treated with Pierce enhanced chemiluminescence substrate (Thermo Scientific, Rockford, IL, USA) and exposed to film.

## 3. Results

### 3.1. Construction of HSV UL21 Mutants by CRISPR/Cas9 Mutagenesis

**Figure 2:**
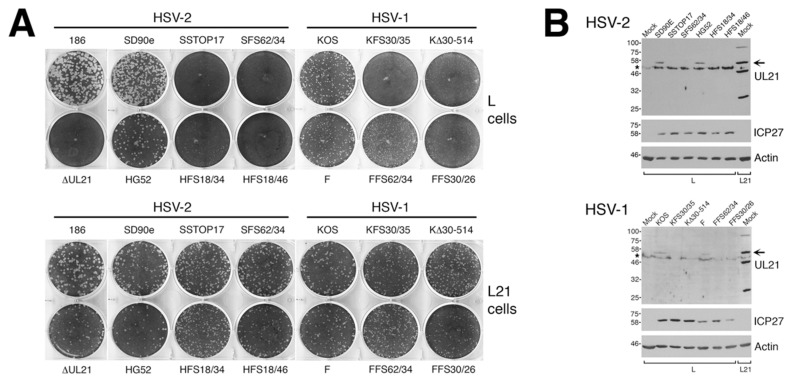
Confirmation of pUL21 deficiency in CRISPR/Cas9 mutants. (**A**) Monolayers of L cells or L21 cells were infected with equivalent numbers plaque forming units of the indicated viruses, overlaid with semisolid medium containing 1% methyl cellulose, then fixed and stained with 70% methanol containing 0.5% methylene blue at 72 hpi. (**B**) Western blot analysis of CRISPR/Cas9 mutants. Whole cell lysates prepared at 24 hpi from L cells infected at a multiplicity of infection (MOI) of 0.1 with the indicated viruses, or from mock infected L or L21 cells, were separated on SDS 8% polyacrylamide gels and transferred to membranes. Membranes were blocked overnight at 4 °C, then probed for the presence of pUL21, ICP27 (infection control), or actin (loading control). Positions of protein molecular weight markers in kDa are indicated on the left. Horizontal arrows indicate the position of full-length pUL21. Asterisks on the left side of the pUL21 panels denote the position of a cellular protein that cross reacts with the pUL21 antiserum.

Studies from multiple laboratories using different strains/species of HSV lacking pUL21 have yielded conflicting results regarding the necessity of pUL21 in HSV replication [1,2,11]. To resolve this discrepancy, we sought to isolate pUL21 deficient viruses from multiple HSV strains using a common methodology that avoided selective pressure. We viewed CRISPR/Cas9 mutagenesis as the most pragmatic approach for isolating these viruses, as it avoided the necessity of establishing a BAC of every strain we wished to study and, instead, required simpler starting materials. Viral DNAs were prepared from four different HSV strains: two strains of HSV-2 (SD90e and HG52) and two strains of HSV-1 (KOS and F). Viral DNAs were co-transfected along with plasmids encoding two different *UL21*-specific gRNAs into complementing cells. By using two gRNAs in these co-transfections, we anticipated introducing deletions into *UL21* that could be readily detected by PCR, thereby simplifying our identification of viruses lacking pUL21. Indeed, we found our CRISPR/Cas9 mutagenesis approach to be straightforward and efficient, yielding a considerable fraction of viruses with a deletion in *UL21* (Figure 1B, 3 out of 4 viruses contain a deletion in *UL21* in the top example, and 5 out of 6 viruses contain a deletion in *UL21* in the bottom example). Complementing cells were used in the initial co-transfections and for propagating recovered viruses, to avoid selective pressure that could result in the appearance of second-site suppressor mutations. A wide variety of lesions were introduced into *UL21*, including premature stop codons, frameshift mutations, and in-frame deletions, all of which would be predicted to give rise to truncated forms of pUL21. Two independent pUL21 deficient viruses were chosen from each strain for further characterization (Figure 1A). Production of pUL21 by L cells (L21 cells) was able to complement the pUL21 deficiency of these viruses (Figure 2A), and pUL21 was not detected in whole cell lysates prepared from L cells infected with these viruses (Figure 2B).

### 3.2. Both HSV-2 and HSV-1 Require pUL21 for Efficient Cell-to-Cell Spread

**Figure 3:**
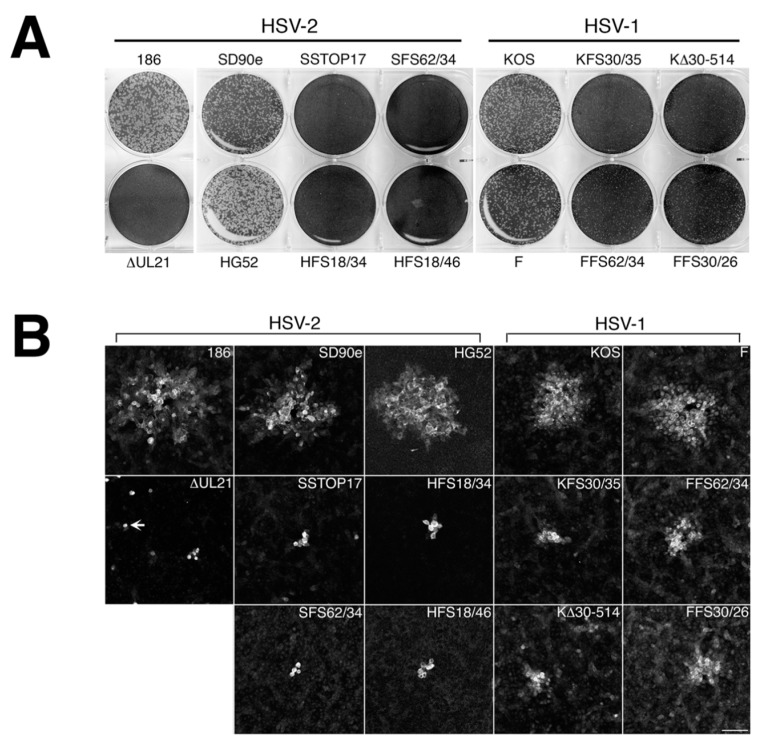

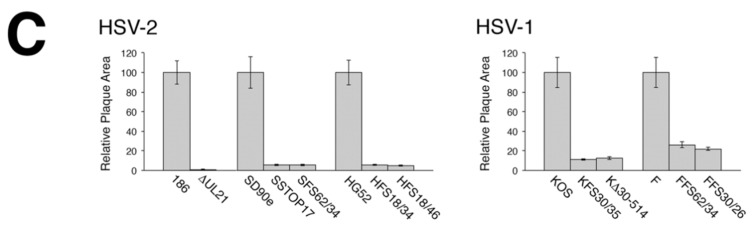
Cell-to-cell spread capabilities of HSV *UL21* deletion mutants. (**A**) Monolayers of Vero cells were infected with equivalent numbers plaque forming units of the indicated virus strains, overlaid with medium containing 1% methyl cellulose, then fixed and stained with 70% methanol containing 0.5% methylene blue at 72 hpi. (**B**) Monolayers of Vero cells growing on glass bottom dishes were infected with the indicated viruses and overlaid with medium containing 1% methyl cellulose. At 24 hpi, infected monolayers were fixed, permeabilized, and then stained for the presence of the HSV kinase Us3. Representative images are shown. Arrow indicates a single infected cell, which were very prominent in Vero cells infected with ∆UL21 at 24 hpi. Scale bar is 100 μm. (**C**) The plaque areas of 40 plaques per virus strain from the experiment depicted in (B) were measured using Image-Pro 6.3 software (Media Cybernetics, Bethesda, MD, USA). Single infected cells were not included in this quantification. Error bars are standard error of the means.

We tested the ability of our panel of pUL21 deficient viruses to form macroscopically visible plaques on non-complementing Vero cells. All HSV-2 pUL21 deficient viruses, including the original *UL21* deletion mutant constructed in strain 186 (∆UL21) [2], failed to yield macroscopically visible plaques on Vero cells at 72 hpi (Figure 3A). All HSV-1 pUL21 deficient viruses yielded macroscopically visible plaques on Vero cells at 72 hpi, however, they appeared smaller than plaques yielded by their parental viruses (Figure 3A).

To quantify the differences in cell-to-cell spread, plaque areas produced by pUL21 deficient viruses on Vero cells at 24 hpi were measured and scored relative to the plaque areas yielded by their parental viruses (Figure 3B,C). All pUL21 deficient viruses had significantly smaller plaque areas than their parental viruses (all *p* values < 0.0001). The difference in plaque size between pUL21 deficient virus and its parental virus was significantly greater for HSV-2 mutants than for HSV-1 mutants (*p* values ranged from <0.0001 to 0.0024). Strain-specific differences in mutant plaque sizes were also noted for both HSV species: SD90e and HG52 mutant plaque sizes were significantly greater than those of the *UL21* mutant derived from strain 186 (all *p* values < 0.0001) and F mutant plaque sizes were significantly greater than those of KOS mutants (*p* values ranged from <0.0001 to 0.0011). Collectively, these findings demonstrate that pUL21 is uniformly required for cell-to-cell spread of HSV. Furthermore, both species-specific and strain-specific differences in the strictness of this requirement have been revealed by this analysis.

### 3.3. Replication Kinetics of HSV-2 and HSV-1 UL21 Deficient Strains

**Figure 4:**
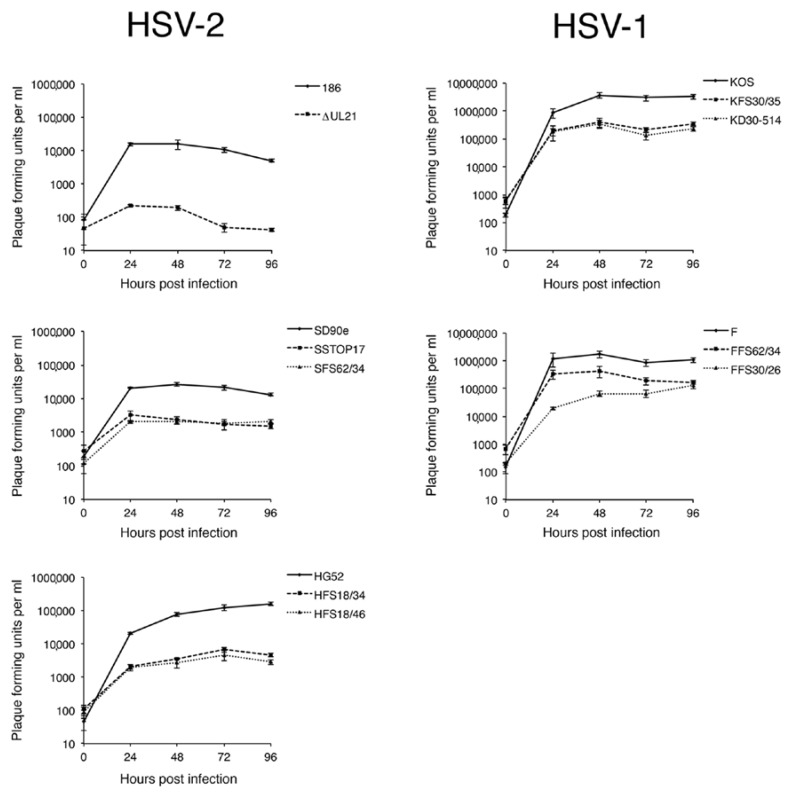
Replication kinetics of HSV *UL21* deletion mutants. Monolayers of Vero cells were inoculated for 1 h at 37 °C with the indicated viruses at an MOI of 0.1, followed by brief treatment with low pH citrate buffer to inactivate virus that had not entered cells. At the indicated time points, cells and medium were harvested together, freeze/thawed twice, sonicated briefly, then centrifuged to remove cellular debris. Clarified supernatants were titrated by plaque assay on monolayers of L21 cells. Each data point represents the average of three independent infections for each virus, titrated in duplicate. Parental HSV strains are indicated with solid lines, and their corresponding *UL21* deletion mutants are indicated with dashed or dotted lines. Error bars are standard error of the means.

To further probe differences between pUL21 deficient strains, a multistep growth analysis in Vero cells was performed (Figure 4). All pUL21 deficient strains showed a significant growth deficiency in comparison to their parental virus (at 72 hpi, *p* values ranged from 0.0164 to <0.0001). The largest growth deficiency observed was for the *UL21* mutant in HSV-2 strain 186 (average of >200-fold at 72 hpi); this value was significantly different from the growth deficiencies observed for all other pUL21 deficient HSV strains at 72 hpi (*p* values ranged from 0.0329 to 0.0002). The ~200-fold growth deficiency of the *UL21* mutant derived from strain 186 in Vero cells was not as severe as previously observed in a similar growth analysis performed in L cells (>1000-fold at 72 hpi; [2]). The growth deficiencies observed for SD90e, HG52, KOS, and F strains lacking pUL21 were all within the 3- to 100-fold range observed in previous studies of KOS and F strains lacking pUL21 [1,5,11].

## 4. Discussion

A necessary foundation for understanding the multiple roles played by pUL21 during HSV infection is to definitively establish whether phenotypic differences exist between HSV species or between HSV strains of the same species. The work detailed in this study provides this foundation. By utilizing CRISPR/Cas9 mutagenesis, we were able to construct multiple HSVs lacking pUL21 in an efficient manner, enabling side-by-side phenotypic analyses. These analyses revealed that HSV-2 has a stricter requirement for pUL21 in cell-to-cell spread of virus than does HSV-1. Strain-specific differences in this phenotype were also noted. Cell-to-cell spread of HSV-2 strain 186 was particularly crippled in the absence of pUL21. In particular, we noted an abundance of ∆UL21 infected Vero cells that failed to spread infection to adjacent cells (Figure 3B, arrow). HSV-2 strain 186 lacking pUL21 also showed a greater deficiency in viral replication in comparison to all other HSV strains lacking pUL21. A previous study from our laboratory demonstrated that HSV-2 strain 186 lacking pUL21 displayed a noticeable defect in nuclear egress [2]. As the results of this study demonstrate that strain 186 lacking pUL21 is an outlier in both cell-to-cell spread of virus and in viral replication kinetics in comparison to other HSV strains lacking pUL21, it will be of considerable interest to investigate whether strain 186 is also an outlier in terms of reliance on pUL21 for nuclear egress.

Strain-specific differences can provide important clues about the function of a viral protein, as well as mechanistic details about a viral process. Thus, investigating the basis of strain-specific differences is a worthwhile endeavor. In this work, we have demonstrated that CRISPR/Cas9 mutagenesis can be used to resolve strain-specific differences. We have also used the methodologies described, herein, to generate deletion mutants of *UL16* in multiple HSV strains as a means of understanding the role of pUL16 in nuclear egress [17]. We anticipate that CRISPR/Cas9 mutagenesis will be valuable for generating viruses carrying deletions in multiple viral genes that will further inform our understanding of alphaherpesvirus nuclear egress.

CRISPR/Cas9 mutagenesis provides an efficient and more broadly accessible means of introducing deletions into alphaherpesvirus strains of interest. Homologous recombination methodologies that were traditionally used to construct alphaherpesvirus mutants had mutant strain recovery efficiencies of less than 1% [15], whereas our CRISPR/Cas9 approach had a mutant recovery efficiency greater than 50% (Figure 1B). More recently, recombineering approaches utilizing select HSV genomes cloned into BACs have been widely used to accurately and efficiently construct HSV mutants. However, the process of constructing a novel BAC is a laborious undertaking [2,18,19,20]. CRISPR/Cas9 technology can be readily and rapidly applied to any HSV strain, and should allow observations made in laboratory strains of alphaherpesviruses to be more readily investigated in clinical isolates.

## Figures and Tables

**Table 1:** Oligonucleotides used to produce HSV-2 and HSV-1 *UL21* gRNAs.

gRNAs	Predicted Nucleotide Cleavage Site ^a^	Top Strand (5′-3′)	Bottom Strand (5′-3′)
HSV-2 *UL21* gRNAs	nt52	5′-CACCGGACGTTGTGTTTTACGTCA-3′	5′-AAACTGACGTAAAACACAACGTCC-3′
	nt183	5′-CACCGACAGGCCCCAAAGACCGCA-3′	5′-AAACTGCGGTCTTTGGGGCCTGTC-3′
HSV-1 *UL21* gRNAs	nt87	5′-CACCGGGCCTACTTTGTGTGCGGG-3′	5′-AAACCCCGCACACAAAGTAGGCCC-3′
	nt184	5′-CACCGACAGGCCCAGACGACCGCG-3′	5′-AAACCGCGGTCGTCTGGGCCTGTC-3′
	nt1540	5′-CACCGCCAGCCGCACGCGGGCCGC-3′	5′-AAACGCGGCCCGCGTGCGGCTGGC-3′

^a^ Nucleotide position in the *UL21* gene targeted by the gRNA.
